# Daily protein-polyphenol ingestion increases daily myofibrillar protein synthesis rates and promotes early muscle functional gains during resistance training

**DOI:** 10.1152/ajpendo.00328.2021

**Published:** 2022-01-17

**Authors:** George F. Pavis, Tom S. O. Jameson, Jamie R. Blackwell, Jonathan Fulford, Doaa R. Abdelrahman, Andrew J. Murton, Nima Alamdari, Catherine R. Mikus, Benjamin T. Wall, Francis B. Stephens

**Affiliations:** ^1^Nutritional Physiology Research Group, Department of Sport and Health Sciences, College of Life and Environmental Sciences, University of Exeter, Exeter, United Kingdom; ^2^Institute of Biomedical and Clinical Sciences, University of Exeter Medical School, University of Exeter, Exeter, United Kingdom; ^3^Department of Surgery, University of Texas Medical Branch, Galveston, Texas; ^4^Sealy Center of Aging, University of Texas Medical Branch, Galveston, Texas; ^5^Beachbody LLC, Santa Monica, California

**Keywords:** deuterated water, hypertrophy, protein-polyphenol, protein synthesis, resistance training

## Abstract

Factors underpinning the time-course of resistance-type exercise training (RET) adaptations are not fully understood. This study hypothesized that consuming a twice-daily protein-polyphenol beverage (PPB; *n* = 15; age, 24 ± 1 yr; BMI, 22.3 ± 0.7 kg·m^−2^) previously shown to accelerate recovery from muscle damage and increase daily myofibrillar protein synthesis (MyoPS) rates would accelerate early (10 sessions) improvements in muscle function and potentiate quadriceps volume and muscle fiber cross-sectional area (fCSA) following 30 unilateral RET sessions in healthy, recreationally active, adults. Versus isocaloric placebo (PLA; *n* = 14; age, 25 ± 2 yr; BMI, 23.9 ± 1.0 kg·m^−2^), PPB increased 48 h MyoPS rates after the first RET session measured using deuterated water (2.01 ± 0.15 vs. 1.51 ± 0.16%·day^−1^, respectively; *P* < 0.05). In addition, PPB increased isokinetic muscle function over 10 sessions of training relative to the untrained control leg (%U) from 99.9 ± 1.8 pretraining to 107.2 ± 2.4%U at *session 10* (vs. 102.6 ± 3.9 to 100.8 ± 2.4%U at *session 10* in PLA; interaction *P* < 0.05). Pre to posttraining, PPB increased type II fCSA (PLA: 120.8 ± 8.2 to 109.5 ± 8.6%U; PPB: 92.8 ± 6.2 to 108.4 ± 9.7%U; interaction *P* < 0.05), but the gain in quadriceps muscle volume was similar between groups. Similarly, PPB did not further increase peak isometric torque, muscle function, or MyoPS measured posttraining. This suggests that although PPB increases MyoPS and early adaptation, it may not influence longer term adaptations to unilateral RET.

**NEW & NOTEWORTHY** Using a unilateral model of resistance training, we show for the first time that a protein-polyphenol beverage increases initial rates of myofibrillar protein synthesis and promotes early functional improvements. Following a prolonged period of training, this strategy also increases type II fiber hypertrophy and causes large individual variation in gains in quadricep muscle cross-sectional area.

## Graphical abstract

**Figure:**
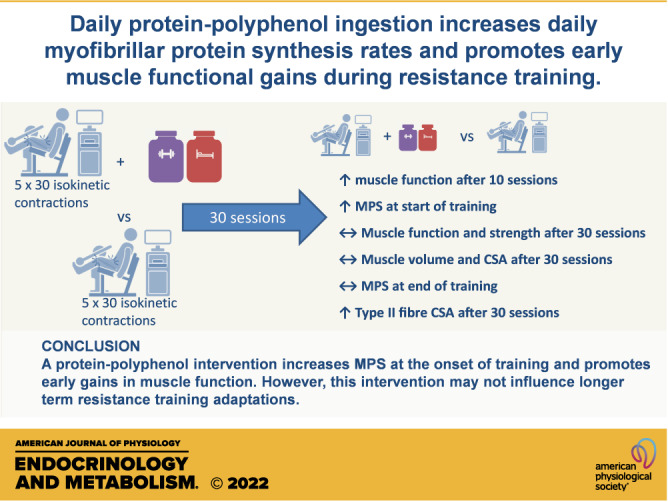

## INTRODUCTION

A period of isokinetic or isotonic resistance-type exercise training (RET) comprising concentric and eccentric loading phases induces skeletal muscle hypertrophy and increases strength (1–5). In both animal (6, 7) and human (8) models of hypertrophy, this expansion in muscle size aligns with greater rates of muscle protein synthesis. For example, in a recent study, Damas et al. (9) demonstrated a remarkable relationship between 48-h integrated myofibrillar protein synthesis (MyoPS) rates and both the increase in muscle fiber (*r*^2^ = 0.83) and *m. vastus lateralis* (*r*^2^ = 0.90) cross-sectional area, when measured after 10 wk of lower limb RET in healthy young men. Interestingly, 48-h integrated MyoPS measured at the start of the RET period was not predictive of subsequent hypertrophy over ∼3 and 10 wk (9), in agreement with prior work documenting an uncoupled relationship between MyoPS at the onset of training and subsequent hypertrophy after 16 wk of RET (10, 11). Given that Damas et al. (9) observed marked myofibrillar disruption (z-band streaming in 60% of fibers), muscle pain (60/100 AU), and a loss of muscle strength (−22%) 48 h after the first RET session, it was suggested that early rates of MyoPS instead reflect a demand for myofibrillar repair following damage rather than net protein accrual. Indeed, supporting this suggestion is that the gains in muscle thickness over the first 3 wk of RET likely reflect damage-related swelling rather than muscle protein accrual (12).

Dietary protein consumed after resistance exercise further increases rates of MyoPS compared with exercise alone (13, 14). Such a strategy also improves muscle strength, lean body mass, and muscle fiber size in young, healthy individuals resistance training for >6 wk (15, 16), in keeping with the theory that greater rates of MyoPS result in greater gains in RET adaptations. We have previously demonstrated that ingestion of a postexercise and prebed protein-polyphenol blend (PPB) increases MyoPS by ∼35% compared with placebo, following recovery from muscle damage (17). Interestingly, and in line with other studies (18, 19), PPB did not further increase MyoPS during the first 3 days of recovery, but did attenuate inflammatory NF-κB signaling, supporting previous work investigating both protein (20) and polyphenols (21) in isolation. Given that PPB also prevented a 30% decline in muscle function 24 h after damaging eccentric and permitted a greater volume of contractile work throughout recovery (17, 22), these data clearly suggest that this combination of protein and polyphenols may accelerate recovery from damage at the onset of training and permit greater gains in muscle function and training volume during the early RET period. Together with evidence that the volume of work performed during RET is one of the primary factors determining MyoPS, strength, and hypertrophic gains (23, 24), this combined protein-polyphenol strategy may subsequently promote training adaptations over prolonged (30 sessions; ∼10 wk) RET.

Taken together, it appears that preventing early muscle damage would enhance early functional adaptations at the onset of a RET program. Therefore, this study aimed to use the same targeted nutritional approach previously shown to improve function following damaging exercise to promote muscle adaptation to prolonged RET and to explore the underlying role of MyoPS. Given that individual, intrinsic factors appear strong determinants of adaptation (25, 26), we employed a supervised unilateral isokinetic training model with measurements made concurrently in the untrained leg to reduce heterogeneity. The model also permitted participants to perform the same number of training sessions and allowed measures of muscle strength (specifically peak isometric and isokinetic torque) and function to be quantified every three sessions, all with high resolution. It was hypothesized that PPB ingestion would accelerate improvements in muscle function during the early (10 sessions; ∼3 wk) training period. Given the tight coupling of MyoPS with hypertrophy at 10 wk of RET, we also hypothesized that this early improvement would be associated with greater posttraining MyoPS rates, and a greater increase in quadriceps muscle volume and fiber CSA.

## METHODS

### Participants

Thirty-two healthy, recreationally active male and female participants volunteered to take part (16 male, 16 female; age: 24 ± 1 yr; BMI: 23.0 ± 0.6 kg·m^−2^). Exclusion criteria were: *1*) diagnosed metabolic or cardiovascular impairment; *2*) self-reported habitual protein intake < 0.8 or > 1.6 g·kg^−1^·day^−1^; *3*) musculoskeletal injury that may impair exercise performance; *4*) engagement in systematic resistance (> 2 times/wk) or endurance (> 6 h/wk) training within 6 mo of participation; and *5*) use of anti-inflammatory medicines or nutritional supplements.

All individuals provided written informed consent at least 24 h after receiving verbal and written explanation of the experimental procedures. This study was approved by the University of Exeter’s Sport and Health Sciences Research Ethics Committee (Ref. No. 171206/B/09) and registered with ClinicalTrials.gov (NCT03918395).

### General Study Design

**Figure 1.**
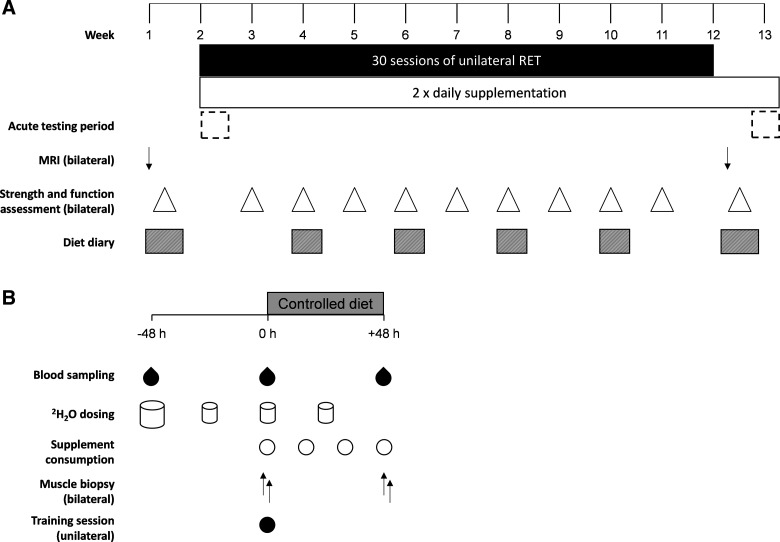
*A*: graphical representation of the entire experimental protocol. One leg was randomly assigned to undergo 30 sessions of resistance-type exercise training with daily consumption of postexercise and prebed protein-polyphenol beverage (PPB; *n* = 15) or maltodextrin placebo (PLA; *n* = 14). MRI scans were obtained of each leg in the rested state pre and posttraining. Muscle strength and function were assessed in both legs following each scan, as well as every three training sessions during training. Habitual dietary intake was recorded pre, during, and posttraining. *B*: schematic of the acute testing period, performed at the onset of training and repeated posttraining. Bilateral muscle biopsies were obtained in the rested state before and 48 h after a single training session. Diet was fully controlled over this 48-h period. Participants consumed 70% ^2^H_2_O at ∼0800 h 2 days prior with 8 × 0.75 mL·kg^−1^ doses consumed every 1.5 h and maintained until the end of the acute measurement window. Daily doses of 0.54 mL·kg^−1^ were provided to maintain enrichment.

Following enrolment, participants were randomly assigned using a double-blind, placebo-controlled, parallel-group design to consume either daily postexercise and prebed protein-polyphenol beverages (PPB) or daily postexercise and prebed placebo beverages (PLA). Participants’ characteristics are shown in Table 1. Before the RET program commenced, height, weight, and habitual dietary intake records were collected. Participants also completed two familiarization visits to become familiarized with all dynamometry testing procedures described herein. Individual settings for the isokinetic dynamometer (Biodex System 3, Biodex Medical Systems, Inc., Shirley, NY) were determined on these visits and used for all subsequent visits. All muscle function and peak torque assessments as well as the RET program were performed on an isokinetic dynamometer. One leg was randomly allocated to undergo RET (T), with leg dominance counterbalanced within each group, whereas the contralateral limb was assigned as the untrained, control (U). Before *session 1* and after *session 30* of the RET program, a bilateral MRI scan of the thighs was performed for the determination of muscle volumes (see *Magnetic Resonance Imaging*) and muscle function and peak isometric and isokinetic torques were measured in both T and U legs. These measurements were repeated every three training sessions throughout the RET program with dietary intake assessed every six sessions (see Fig. 1 for a schematic overview). In addition, muscle biopsy and venous blood samples were obtained from a subset of participants (PLA: *n* = 10; PPB: *n* = 11).

**Table 1.** Participant characteristics

	PLA	PPB
	(*n* = 14)	(*n* = 15)
Sex, male:female	7:7	7:8
Age, yr	25 ± 2	24 ± 1
Body mass, kg	67.6 ± 2.5	65.5 ± 3.5
Height, cm	168 ± 3	170 ± 3
BMI, kg·m^−2^	23.9 ± 1.0	22.3 ± 0.7
Baseline function, J	U: 2,172 ± 180 T: 2,204 ± 173	U: 2,413 ± 216 T: 2,418 ± 232
Baseline peak isometric torque, n·m	U: 185 ± 10 T: 184 ± 13	U: 211 ± 21 T: 204 ± 21
Baseline peak isokinetic torque, n·m	U: 139 ± 12 T: 132 ± 12	U: 153 ± 16 T: 150 ± 15

Values represent means ± SE. Between-group comparisons *P* > 0.05. BMI, body mass index; PLA, maltodextrin placebo group; PPB, postexercise and prebed protein-polyphenol group; T; trained leg; U, untrained leg.

### Muscle Function and Peak Torque Assessment

Participants visited the laboratory for pretraining measures of knee extensor muscle function, peak isometric and isokinetic torque, and muscle volume (see *Magnetic Resonance Imaging*) performed in the fasted state at ∼08:30 after abstaining from strenuous physical activity, alcohol, and or analgesic medication for 48 h and caffeine for 24 h.

Peak isometric torque was assessed by maximal isometric voluntary contraction of the knee extensor muscles. After a task-specific warm-up consisting of 3 s isometric contractions at 50% (×2), 75% (×1), and 90% (×1) of perceived maximal effort, participants performed 3 × 3 s maximal isometric voluntary contractions at 75° knee flexion separated by 60 s rest (full extension corresponding to 0°). Next, participants completed a warm-up set of five submaximal isokinetic contractions of the knee extensor muscles. Then, following a 60 s rest, peak isokinetic torque was determined over three maximal isokinetic contractions. Finally, for the assessment of muscle function, participants then rested for a further 60 s before performing 30 maximal isokinetic contractions. For isokinetic torque and muscle function assessments, knee joint range of motion was set at 80° equidistant to full flexion and full extension and each contraction was performed at 75°·s^−1^.

Torque was recorded over each isometric and kinetic contraction and sampled at 100 Hz with an analog-to-digital converter (Powerlab 4/26, ADInstruments, Dunedin, New Zealand) and accompanying software (Labchart v. 8, ADInstruments). Peak isometric and isokinetic torque (n·m) were determined from the peak torque achieved during any of the three contractions, and muscle function (J) was defined as total isokinetic work calculated from the area under the torque-time graph over the first 30 maximal repetitions in each leg, as previously described (17, 22). Participants received verbal encouragement and were instructed to push as hard as possible for each contraction, which were separated by 60 s rest.

All measurements were carried out in both T and U legs and repeated as aforementioned posttraining. Additional bilateral measures of muscle function and peak torque were made 48 h after every three training sessions to provide detailed resolution on the time-course of training adaptations.

### Magnetic Resonance Imaging

Magnetic resonance imaging (MRI) for determination of thigh and quadriceps muscle volume was determined at ∼0900, immediately before the pre and posttraining function and peak torque testing, as previously described (27). Briefly, a 1.5-T MRI scanner (Intera, Philips, The Netherlands) was used to take axial plane images over the full length of the femur. A T1-weighted 3-D turbo spin echo sequence was used (field of view 500 × 500 mm, reconstructed matrix 512 × 512 mm, echo time 15 ms, repetition time 645 ms, slice thickness 5 mm, slice gap 5 mm) while the subject remained supine. A four-element sense body radiofrequency coil surrounded both thighs. During the pretraining scan, a specified distance from a bony landmark (femoral condyle) on the dominant leg in the frontal plane was used to center the axial plane images. This same distance was used on all subsequent MRI scans to ensure the axial images were in the same location along the length of the thigh on all scans.

Slicer software [v. 4.10.0; (28)] was used to analyze the images obtained in the axial plane and calculate the muscle volumes. First, the length of the femur between the lateral condyle and the greater trochanter was determined from the obtained images as previously described (29), and the top 25% of slices (from the greater trochanter working distally) and bottom 25% of slices (from the lateral condyle working proximally) were excluded so that only the middle 50% region of the length of the femur area was used for automated thresholding. The anatomical cross-sectional area (aCSA) of the thigh muscle on each slice was calculated by using the thresholding function in Slicer, with manual erasing applied as required to ensure only muscle tissue was thresholded (i.e., excluding bone, adipose tissue and skin). Quadriceps muscle volume was determined by manually erasing the thresholding of nonquadriceps muscles (i.e., hamstring and adductor muscles) from the thresholding of thigh muscle on each slice. Muscle volume was calculated using a previously published method (29) where the total muscle cross-sectional area for all slices was calculated and multiplied by the slice thickness plus the distance between slices (linear interpolation; in this case a total of 1 cm, 5 mm slice thickness, 5 mm slice gap), summarized by the following equation:

(*1*) $$\mathrm{Muscle}\;\mathrm{volume}\;\left(\text{c}{\text{m}}^{3}\right)=\underset{\mathrm{aCSA}}{\sum }�(\mathrm{slice}\;\mathrm{thickness}+\mathrm{slice}\;\mathrm{gap}).$$

Proximal, central, and distil thigh and quadriceps muscle aCSA were obtained from slices at the mid femur (central) and at 50% of the distance from the mid femur to both the most proximal and distil edge of the region of interest (∼5 cm proximal and distil from the mid femur depending on femur length). The same experimenter (T.S.O.J.) analyzed all images.

### Acute Testing Period

Participants were instructed to avoid strenuous physical activity, alcohol, and anti-inflammatory or analgesic medication for 48 h before and throughout a 48 h acute testing period commencing on the morning of both the 1st and 31st training sessions (Fig. 1). Following a > 10-h overnight fast, participants arrived at the laboratory and muscle soreness was assessed upon standing from a seated position using a 100-mm visual analog scale (VAS) (30). Next, a subset of participants provided a venous blood sample from the antecubital vein and biopsy sample from the *m. vastus lateralis* of each leg. All participants then commenced a supervised training session (either 1st session pretraining or 31st session posttraining). Once completed, participants consumed their respective postexercise beverage (see *Nutritional Intervention*) and left the laboratory. These procedures were repeated 48 h later to finish the acute testing period. When performed pretraining, participants remained in the laboratory to complete their second training session. After the acute testing period posttraining, participants completed their enrolment in the study.

Dietary intake was fully controlled during the acute testing period with participants’ energy requirements calculated as the basal metabolic rate (estimated via the Henry equations) (31), multiplied by an activity factor of 1.6 to maintain energy balance, as evidenced previously (17). In line with our previous work, dietary protein was clamped at 1.2 g·kg body mass^−1^·day^−1^ (17, 22). No other energy-containing food or beverages were permitted over this time, whereas water and noncaloric drinks were allowed ad libitum. Compliance with the nutritional intervention was assessed via dietary records, returned food containers and daily communication with the participants.

Forty-eight hours before each acute testing period, participants electing to undergo the biopsy procedure visited the laboratory to commence the deuterated water dosing protocol as previously described (17, 22). Briefly, this consisted of 1 loading day to enrich the body water pool to ∼0.6% using 6 mL·kg body mass^−1^ of 70% ^2^H_2_O (Cambridge Isotopes Laboratories, Andover, MA, separated into eight equal doses. This was followed thereafter by 3 maintenance days, where participants consumed 0.54 mL·kg^−1^ once daily upon waking.

### Resistance-Type Exercise Training Program

Participants performed exactly 30 sessions of supervised resistance-type exercise during the RET program, with the aim of performing 3 sessions/wk. Although training was continuous, the first 10 sessions comprised the early training period, with *sessions 10–30* referred to collectively as the late training period. Each training session consisted of five sets of 30 maximal isokinetic knee extensor contractions alternating between concentric and eccentric contraction modalities (3 sets of concentric contractions and 2 sets of eccentric contractions/session), which would be expected to induce significant hypertrophy of the *m. vastus lateralis* (3) and mimic a conventional resistance-type muscle contraction involving both a concentric and eccentric contraction phase. As with isokinetic torque and muscle function assessments, RET was performed on an isokinetic dynamometer with the knee joint range of motion set at 80° equidistant to full flexion and full extension and each contraction performed at 75°·s^−1^. Verbal encouragement was provided throughout each set, and each set was separated by 120 s passive rest. By using an identical number or repetitions, performed over an identical range of motion at a maximal intensity, each participant was theoretically exposed to an identical training stimulus thereby providing a highly controlled RET model with which to investigate the surplus effect of a nutritional intervention. Moreover, we quantified the isokinetic work performed during each repetition, providing us with set-by-set and session-by-session insight into temporal changes in both concentric and eccentric training volume. Mean duration for the RET program was 73 ± 2 and 73 ± 3 days for PLA and PPB groups, respectively, with sessions separated by 2 ± 0 days for both groups (all group differences *P* > 0.05).

### Nutritional Intervention

The nutritional intervention began immediately after training *session 1* and continued throughout the RET program until the completion of the full study protocol. A postexercise beverage was provided to participants after completing each training session. Beverages were made up from sachets that were coded but otherwise identical in appearance to ensure double-blinding of both the researchers and participants. These contained either a commercially available postexercise supplement (Beachbody Performance Recover, Beachbody LLC, Santa Monica, CA), providing 20 g total protein (from a blend of whey, pea, and casein; of which 2.4 g is leucine; Supplemental Table S1; all Supplemental Material is available at https://doi.org/10.6084/m9.figshare.17892506), 10 g total carbohydrate, and 650 mg pomegranate extract for participants in PPB treatment, or a taste- and color matched isocaloric, maltodextrin placebo for participants in PLA, added to 225 mL of water. Afterward, an additional 50 mL was added to the bottles and given to the participants to ensure that all the contents were consumed.

Participants were provided with additional sachets to consume on nontraining days at home in the same manner. In addition, participants were provided with a different prebed beverage to consume within 30 min of going to bed each night. The prebed PPB beverage contained 18 g of protein (of which 1.9 g is leucine; Supplemental Table S1) from micellar casein and 480 mg of tart cherry extract, or a taste- and color matched isocaloric, maltodextrin beverage for participants in PLA. All drinks were well tolerated and no adverse effects were reported during or after the experimental period. Adherence to the nutritional intervention was assessed using a diary to record the time of consumption. Adherence was 98 ± 1% in PLA and 99 ± 0% in PPB, with no differences between groups.

### Habitual Dietary Intake

Before and after the RET program, participants were instructed to record a 3-day weighted, habitual food diary to include two weekdays and one weekend day. Further 2-day habitual food diaries were completed every six training sessions (approximately every 2 wk) to track habitual dietary intake throughout training. Habitual energy and macronutrient intakes were calculated from these food diaries using online licensed software (Nutritics, Swords, Dublin, Ireland). In line with our exclusion criteria, participants were instructed to adjust their protein intake accordingly if the 2-day diary indicated that protein intake excluding supplements was <0.8 or >1.6 g·kg^−1^·day^−1^.

### Blood and Muscle Sampling

Fasted venous blood samples were collected immediately before the deuterated water dosing protocol and immediately before each muscle biopsy. Samples were collected from the antecubital vein via venipuncture technique into lithium heparin containers, which were immediately centrifuged at 2,850 *g* for 10 min at 4°C. Plasma was aliquoted, frozen at −20°C, and then transferred to storage at −80°C for further analysis.

Muscle biopsies were obtained under local anesthesia (2% lidocaine) from the midsection of the *m. vastus lateralis* by the Bergström needle technique modified for suction (32). All samples were rapidly dissected of visible fat and connective tissue, frozen in liquid-nitrogen-cooled isopentane, and stored at −80°C until subsequent analysis.

### Plasma and Muscle Enrichments

Plasma hydrogen isotope ratios (^2^H/^1^H) were determined by injecting samples into a high-temperature conversion elemental analyzer (TCEA Flash 2000, Thermo Fisher Scientific, Waltham, MA) coupled to an isotope ratio mass spectrometer (IRMS; Delta V, Thermo Fisher Scientific). Samples were run in triplicate. Raw isotope ratio values were normalized to in-house reference materials calibrated to Vienna Standard Mean Ocean Water (VSMOW).

The enrichments of [^2^H]-alanine in the myofibrillar fraction of skeletal muscle tissue samples were determined as previously described (17, 22). Briefly, myofibrillar protein fractions were isolated from ∼50 mg wet weight muscle tissue. Tissue was homogenized and centrifuged, then separated from the sarcoplasmic supernatant. The resultant pellet was solubilized in 0.3 M NaOH and separated from the insoluble collagen fraction by centrifugation. Myofibrillar proteins were precipitated with the addition of 1 M perchloric acid, washed twice in 70% ethanol, and hydrolyzed in 6 M HCl at 110°C for 24 h. Amino acids were purified using cation exchange resin columns (100–200 mesh; H^+^ form; Dowex 50WX8; Sigma-Aldrich Company Ltd) and dried under vacuum before storage at −20°C.

Purified amino acids were derivatized to their tert-butyl-dimethylsilyl (TBDMS) esters (33) and injected into a Delta V Advantage IRMS (Thermo Fisher Scientific) fitted with a Trace 1310 gas chromatograph. Amino acids were separated using a 30 m × 0.25 mm ID × 0.25 μm film DB-5 capillary column (Agilent Technologies, Santa Clara, CA; temperature program: 110°C for 1 min; 10°C·min^−1^ ramp to 180°C; 5°C·min^−1^ ramp to 220°C; 20°C·min^−1^ ramp to 300°C; hold for 2 min), before undergoing pyrolysis and analysis via IRMS. The enrichment of tracer was measured by monitoring ion masses 2 and 3 to determine the ^2^H/^1^H ratios of myofibrillar protein-bound [^2^H]-alanine. A series of known standards were applied to assess the linearity of the mass spectrometer.

### Myofibrillar Protein Synthesis

Myofibrillar protein fractional synthetic rates (myoFSR) were calculated to quantify MyoPS, based on the incorporation of [^2^H]-alanine into myofibrillar protein and mean plasma deuterium enrichment between muscle biopsy time points, which has been previously validated for use as a surrogate precursor pool (34). Plasma enrichment was corrected by a factor of 3.7, based on the deuterium labeling of alanine (35). MyoFSR was calculated using the following precursor product equation:

(*2*) $$\mathrm{myoFSR}\;\left(\%\cdot {\mathrm{day}}^{-1}\right)\;=\left(\frac{E_{m2}-E_{m1}}{3.7\;\times \;E_{p}\;\times \;t}\right)\;\times \;100,$$

where *E_m_*_1_ and *E_m_*_2_ are the myofibrillar protein-bound alanine enrichments expressed as mole per cent excess (MPE), *E_p_* represents mean precursor enrichment between given time points, and *t* is the time between the corresponding biopsies for which *FSR* is calculated, expressed as days.

### Immunohistochemistry

Muscle biopsy samples collected at 0 h during the acute testing period pre and posttraining were embedded in optimal cutting temperature compound to align fibers perpendicular to the horizontal surface. Microtome-cryostat sections (7 μm thick) were obtained at −22°C and mounted on glass slides, which were then visually inspected with light microscopy to ensure correct alignment and true cross-sections. The same researcher (G.F.P.) mounted all sections and was blinded to participant grouping. Slides were subsequently defrosted to room temperature and fixed in 4% paraformaldehyde in phosphate-buffered saline (PBS) for 15 min. After washing in PBS, a PBS-based blocking solution (5% fetal bovine serum, 2% bovine serum albumin, 2% goat serum, 0.2% Triton X-100, and 0.1% sodium azide) was applied for 60 min followed by overnight incubation at 4°C with mouse anti-Pax7 (isotype MIgG1; 1:40 in PBS; Developmental Studies Hybridoma Bank, Iowa City, IA; RRID: AB_528428). The following morning, slides were washed in PBS and incubated for 2 h with mouse anti-laminin (MIgG2a; 1:100; Developmental Studies Hybridoma Bank; RRID: AB_2134060) and mouse anti-myosin heavy chain type I (MIgM; 1:100; Developmental Studies Hybridoma Bank; RRID: AB_528384) in PBS. After washing, slides were incubated for 2 h in PBS containing goat anti-mouse IgG1 (1:1,000; Alexa Fluor 488, Invitrogen, Paisley, UK; RRID: AB_2535764), goat anti-mouse IgG2a (1:250; Alexa Fluor 568, Invitrogen; RRID: AB_2535773), and goat anti-mouse IgM (1:100; Alexa Fluor 647, Abcam PLC, Cambridge, UK; RRID: AB_2893175), with 42 mM DAPI. After a final washing step, slides were mounted with cover glass with Mowiol (Sigma-Aldrich Company Ltd).

All images were captured digitally (LAS X software; Leica Microsystems GmbH, Wetzlar, Germany) using a Leica DMi8 S widefield fluorescence microscope (Leica Microsystems GmbH) coupled to a Hamamatsu C11440-22C camera (Hamamatsu Photonics, Shizuoka, Japan) at ×20 magnification. Epifluorescence signal was recorded by using excitation filters for DAPI (400 nm), laminin (Texas Red, 540–580 nm), PAX7 (FITC, 465–495 nm), and MHC-I (Y5, 620–650 nm). Image processing and quantitative analysis were conducted by the same experimenter (G.F.P.) using the open-source Fiji software platform (36) with the MuscleJ plug-in (37) to quantify satellite cell number, fiber type, and the mean fiber cross-sectional area (fCSA). G.F.P. visually inspected all automatically selected fibers to ensure they were cross-sectional. As a measure of fiber circularity, form factors were calculated by the MuscleJ plug-in using the following formula:

(*3*) $$\frac{4\pi \times \mathrm{CSA}}{{\mathrm{perimeter}}^{2}}.$$

Fibers were not analyzed if the form factor was below 0.4. Following quantification, regions of interest were manually verified, and any misidentified fibers were removed from the subsequent analysis. Following this correction, 329 ± 31 fibers were analyzed per section (of which 139 ± 17 were type I fibers; 190 ± 16 were type II fibers).

### Muscle Protein and DNA Content

Muscle wet weight (19.2 ± 0.8 mg) was determined on a precision microbalance before freeze-drying for 48 h. The samples were then powdered by hand, separating muscle from any remaining blood and connective tissue, and aliquoted for further analysis. Freeze-dried muscle powder (3.5 ± 0.1 mg) was reweighed and analyzed for protein and DNA content according to Forsberg et al. (38). Briefly, powder was precipitated in 0.5 mL PCA on ice with regular vortexing. Following centrifugation, the supernatant was removed and the pellet was washed twice with 500 μL PCA, before being dissolved in 1 mL 0.3 M KOH. Protein content was then determined in aliquots in duplicate by colorimetric assay (DC protein assay, Bio-Rad Laboratories Inc., CA). DNA was precipitated from the remaining sample using 1.2 M PCA on ice for 30 min before centrifugation. The resultant pellet was hydrolyzed with 250 μL PCA for 1 h at 70°C, before determining DNA content by the diphenylamine reaction (39).

### Statistical Analyses

Baseline characteristics between groups were investigated using a Student’s independent *t* test. A two-way mixed model ANOVA was used to identify differences between pretraining habitual diet versus controlled diet (diet and group factors). Two-way mixed model ANOVAs with time and group factors were used to assess changes in habitual diet throughout training, total work performed throughout training, muscle function and peak torque, thigh and quadriceps volume and CSA, fCSA, fiber type proportion, and satellite cell content per muscle fiber. A three-way ANOVA was used to identify improvements in work performed by contraction type (i.e., eccentric vs. concentric phases), expressed as the percentage of work performed in *session 1* (time, group, and contraction type factors). Interaction effects were followed up using two-way mixed model ANOVAs. Correlation analyses were performed using Pearson’s product moment correlation between training adaptations and MyoPS for participants where full sets of data were obtained. Correlation coefficients |r| < 0.2 were considered small; 0.2 < |r| < 0.7, moderate; and |r| > 0.7, high. For muscle function and measures of hypertrophy, standard deviation of individual responses was calculated from standard deviation of change scores for training outcomes using spreadsheets available from Hopkins (40) as described previously (41). Values were converted into standardized (Cohen) units with thresholds for interpreting the magnitude of effects halved such that 0.1, 0.3, 0.6, 1.0, and 2.0 represent small, moderate, large, very large, and extremely large effects (41). Significance level was set as *P* < 0.05, whereas trends were noted if 0.05 < *P* < 0.10. Unless otherwise specified, data presented as means ± SE, and where possible, presented corrected to the untrained control leg (%U). Uncorrected data are available in Supplemental Tables S2 and S3.

## RESULTS

Participant characteristics for each treatment group are shown in Table 1. There were no baseline differences in any measured variable between groups. Three subjects did not complete the full experimental protocol; two due to personal circumstances and one due to time commitments, thus data from 29 participants were included in the final data set (PLA: *n* = 14; PPB: *n* = 15). Upon completion of the study, participants were asked whether they could identify the intervention they had been assigned to; four participants in PLA and eight participants in PPB had correctly identified their group, whereas the remaining eight participants in PLA and five participants in PPB either did not know or guessed incorrectly. Over the RET program, body weight and BMI did not change in either group.

### Dietary Intake

There were no group differences in self-reported, habitual diet assessed pretraining. Over the duration of the study, total daily energy, and carbohydrate and fat intake expressed both as absolute values and relative to body mass, was unchanged over time in PLA and was not affected by PPB intervention (Tables 2 and 3). Absolute and relative protein intake was unchanged over time in PLA (Table 2), but was significantly greater than pretraining at all-time points with PPB intervention (interaction *P* < 0.001). As contribution of daily energy intake, protein intake decreased from pre to posttraining in PLA (post hoc *P* < 0.05), but was significantly greater following PPB intervention at all timepoints versus pretraining (interaction *P* < 0.001*;* post hoc *P* < 0.05). Although carbohydrate consumption proportional to total energy intake was unchanged, the contribution of fat decreased in PLA (time effect *P* < 0.05), with no further effect of PPB.

**Table 2.** Habitual diet pre, during, and post 30 sessions of resistance-type exercise training in a group randomized to consume a daily postexercise and prebed maltodextrin placebo (PLA) nutritional intervention

	PLA					
	Pre	*Session 7*	*Session 13*	*Session 19*	*Session 25*	Post
Energy, MJ·day^−1^	8.7 ± 0.9	9.8 ± 0.8	9.5 ± 0.8	9.1 ± 0.5	9.7 ± 0.6	9.1 ± 0.6
Protein, g·kg bm^−1^·day^−1^	1.4 ± 0.2	1.5 ± 0.2	1.2 ± 0.1†	1.2 ± 0.1†	1.4 ± 0.1†	1.3 ± 0.1
Protein, g·day^−1^	96 ± 12	97 ± 16	80 ± 9	83 ± 7	94 ± 7	85 ± 7
Carbohydrates, g·day^−1^	231 ± 24	273 ± 18	273 ± 27	272 ± 18	272 ± 21	266 ± 20
Fat, g·day^−1^	83 ± 10	91 ± 9	87 ± 10	77 ± 4	92 ± 8	80 ± 6
Protein, En%	19 ± 1	16 ± 2†	14 ± 1†	15 ± 1†	16 ± 1†	16 ± 1*†
Carbohydrates, En%	45 ± 2	48 ± 2	48 ± 2	50 ± 1	47 ± 2	49 ± 2
Fat, En%	35 ± 2	34 ± 2	34 ± 2	32 ± 1	35 ± 2	33 ± 1

Values represent means ± SE. A two-way ANOVA was used to detect differences over time and compared with PPB (Table 3). *P < 0.05 significantly different to pre. †*P* < 0.05 significantly different to corresponding value in PPB (Table 3). PLA, maltodextrin placebo group; PPB, postexercise and prebed protein-polyphenol group.

**Table 3.** Habitual diet pre, during, and post 30 sessions of resistance-type exercise training in a group randomized to consume a daily postexercise and prebed protein-polyphenol (PPB) nutritional intervention

	PPB					
	Pre	*Session 7*	*Session 13*	*Session 19*	*Session 25*	Post
Energy, MJ·day^−1^	8.3 ± 0.8	8.8 ± 0.8	8.8 ± 0.7	7.8 ± 0.6	8.0 ± 0.6	9.2 ± 0.6
Protein, g·kg bm^−1^·day^−1^	1.2 ± 0.1	1.8 ± 0.1*	1.7 ± 0.1*†	1.7 ± 0.1*†	1.7 ± 0.1*†	1.8 ± 0.1*
Protein, g·day^−1^	77 ± 8	116 ± 9*	106 ± 6*	108 ± 7*	107 ± 6*	115 ± 8*
Carbohydrates, g·day^−1^	232 ± 22	235 ± 19	233 ± 22	211 ± 15	219 ± 20	247 ± 15
Fat, g·day^−1^	84 ± 11	76 ± 11	81 ± 8	65 ± 8	64 ± 7	82 ± 7
Protein, En%	16 ± 1	23 ± 2*†	21 ± 1*†	24 ± 1*†	23 ± 1*†	21 ± 1*†
Carbohydrates, En%	47 ± 2	45 ± 2	44 ± 2	46 ± 2	46 ± 2	45 ± 1
Fat, En%	37 ± 2	31 ± 2	34 ± 2	30 ± 2	30 ± 2	33 ± 1

Values represent means ± SE. A two-way ANOVA was used to detect differences over time and compared with PLA (Table 2). *P < 0.05 significantly different to pre. †*P* < 0.05 significantly different to corresponding value in PLA (Table 2). PLA, maltodextrin placebo group; PPB, postexercise and prebed protein-polyphenol group.

### Muscle Soreness

Baseline muscle soreness measured before the first training session was 4 ± 2 and 3 ± 1 mm in PLA and PPB, respectively. The onset of training significantly increased muscle soreness (*P* < 0.001), such that before *session 2*, 48 h after the first training session, muscle soreness had increased to 7 ± 2 and 9 ± 2 mm in PLA and PPB (*P* < 0.001), respectively. Before *session 3*, muscle soreness was significantly lower and had returned to baseline (2 ± 1 and 4 ± 1 mm in PLA and PPB, respectively; *P* < 0.001). Intervention with PPB did not affect muscle soreness compared with PLA.

### Volume of Work during Training

**Figure 2.**
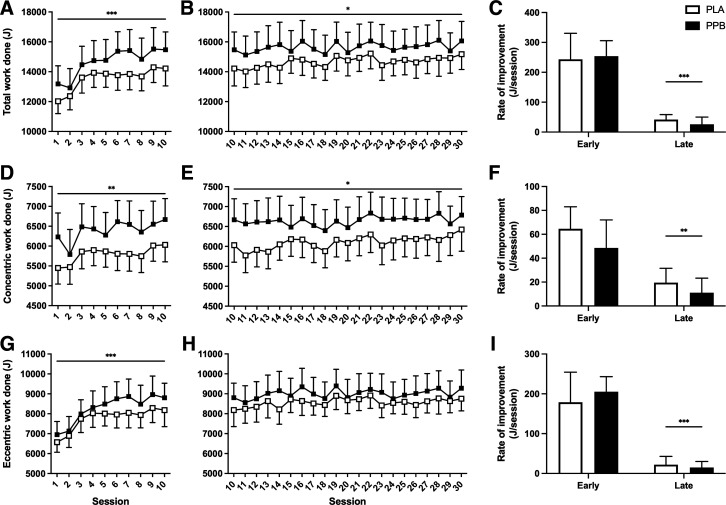
Total (*A–C*), concentric (*D–F*), and eccentric (*G–I*) work over early (*sessions 1–10*; *left*) and late (*sessions 10–30*; *middle*) periods of unilateral resistance-type exercise training. The *right* panels present a comparison of rate of improvement between early and late periods. Participants performed 5 sets of 30 repetitions of alternating concentric and eccentric maximal, isokinetic, knee extensor contractions. A postexercise and prebed protein-polyphenol (PPB; filled symbols; *n* = 15) or maltodextrin placebo (PLA; open symbols; *n* = 14) nutritional intervention was provided daily. Values are means ± SE. Statistical analysis performed with separate two-way ANOVA. Significant main effects of training denoted by *P < 0.05, **P < 0.01, ***P < 0.001.

Training *session 10* marked the end of the early phase of training and was performed 3.0 ± 0.1 wk after onset of RET. Total work performed during training increased from *session 1* to *session 10* and was unaffected by nutritional intervention (19.1 ± 6.6% in PLA; 20.6 ± 4.3% in PPB; time effect *P* < 0.001; Fig. 2*A*). Concentric work increased by 11.6 ± 3.3% and 10.0 ± 4.1% (time effect *P* < 0.01; Fig. 2*D*), and eccentric work increased 27.7 ± 12.8% and 30.9 ± 6.0% (time effect *P* < 0.001; Fig. 2*G*) in PLA and PPB, respectively, with no effect of nutritional intervention.

**Figure 3.**
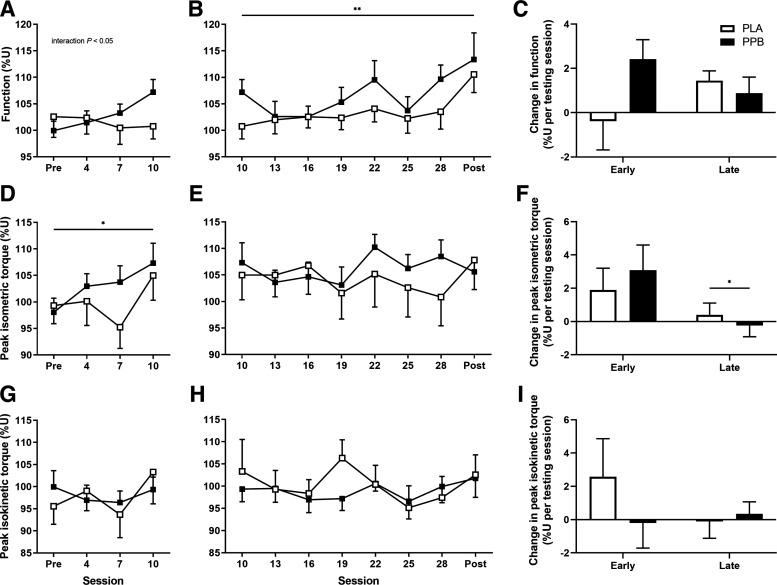
Knee extensor muscle function (*A–C*), peak isometric torque (*D–F*), and peak isokinetic torque (*G–I*) over early (*sessions 1–10*; *left*) and late (*sessions 10–30*; *middle*) periods of unilateral resistance-type exercise training, expressed relative to the untrained leg (%U). The *right* panels present a comparison of rate of improvement between early and late periods. Muscle function was defined as total work performed over 30 maximal, voluntary, isokinetic, concentric contractions. Peak isometric torque measured during 3 × 3 s maximal voluntary isometric contractions (MVC). Peak isokinetic torque was measured during three maximal, voluntary, isokinetic, concentric contractions. A postexercise and prebed protein-polyphenol (PPB; filled symbols; *n* = 15) or maltodextrin placebo (PLA; open symbols; *n* = 14) nutritional intervention was provided daily. Values are means ± SE. Statistical analysis performed with separate two-way ANOVA. Significant main effects of training denoted by *P < 0.05, **P < 0.01.

During the latter stage of training, total work performed increased a further 9.4 ± 3.3% in PLA and 5.4 ± 3.4% in PPB, with no effect of nutritional intervention (Fig. 3*B*). Although concentric work increased a further 6.4 ± 3.1% and 3.9 ± 2.8% in PLA and PPB (time effect *P* < 0.05; Fig. 3*E*), the additional rise in eccentric work was not significant (PLA: 11.3 ± 7.1%; PPB: 7.1 ± 5.2%; time effect *P* = 0.12; Fig. 3*H*). The mean change in work performed per session was significantly greater during the early versus the late phase of training (total work *P* < 0.001; concentric work *P* < 0.01; eccentric work *P* < 0.001). PPB did not influence the rate of improvement in training work at any stage.

Cumulative work performed over the first week (7.3 ± 0.3 days following initial training session) was unaffected by PPB intervention (PLA: 52.9 ± 4.4 kJ; PPB: 54.4 ± 4.4 kJ). By *session 10*, PLA and PPB had performed similar volumes of work (137.7 ± 11.5 kJ; 144.1 ± 11.7 kJ).

### Muscle Function, Peak Isometric Torque, and Peak Isokinetic Torque

No baseline differences were observed in muscle function, peak isometric, or isokinetic torque either between legs or between groups (Table 1). In PLA, muscle function was unaffected by RET over the early phase (Fig. 3*A*), from 102.6 ± 3.9%U pretraining to 100.8 ± 2.4%U at *session 10*. However, PPB increased function over this time (time × group interaction *P* < 0.05), from 99.9 ± 1.8%U to 107.2 ± 2.4%U at *session 10* (post hoc pre vs. *session 10 P* = 0.067). Peak isometric torque increased from pretraining to *session 10* in PLA (99.3 ± 3.4 to 105.0 ± 4.7%U, pre to *session 10*, respectively; Fig. 3*D*) and to a similar extent in PPB (98.0 ± 2.7 to 107.2 ± 2.4%U; pre to *session 10*, respectively; time effect *P* < 0.05). Peak isokinetic torque (Fig. 3*G*) was unaffected by training or nutritional intervention over the early phase.

During the late training period, muscle function changed over time (time effect *P* < 0.01; Fig. 3*B*). Peak isometric (Fig. 3*E*) and isokinetic torque (Fig. 3*H*) were unaffected by training and nutritional intervention over this time.

When comparing pre to posttraining outcomes, 30 sessions of RET significantly increased muscle function, from 102.6 ± 3.9 to 110.6 ± 3.4%U pre to posttraining in PLA (time effect *P* < 0.01). A similar increase was observed in PPB (99.9 ± 1.8 to 113.5 ± 5.0%U pre to posttraining), revealing no additional effect of nutritional intervention on muscle function. Training increased peak isometric torque from 99.3 ± 3.4 to 107.8 ± 5.6%U pre to posttraining in PLA. PPB did not affect this gain, increasing from 98.0 ± 2.7 to 105.6 ± 2.1%U (time effect *P* < 0.05). Peak isokinetic torque was unaffected by training or nutritional intervention.

### Myofibrillar Protein Synthesis

Following consumption of deuterated water pretraining, mean plasma deuterium enrichment was 0.72 ± 0.02 and 0.68 ± 0.02 atom percent excess (APE) in PLA and PPB, respectively, with no differences over time or between groups (Table 4). When repeated posttraining, mean plasma deuterium enrichment was 0.68 ± 0.02 and 0.64 ± 0.01 APE with no differences observed over time or between groups.

**Table 4.** Plasma ^2^H and myofibrillar protein-bound [^2^H]-alanine enrichments over the acute testing periods pre and posttraining

	PLA		PPB	
	0 h	48 h	0 h	48 h
Pretraining				
Plasma ^2^H enrichment (APE)	0.710 ± 0.031	0.726 ± 0.028	0.675 ± 0.019	0.680 ± 0.026
U leg [^2^H]-alanine enrichment (MPE)	0.151 ± 0.024	0.231 ± 0.027 ***	0.112 ± 0.010	0.204 ± 0.016 ***
T leg [^2^H]-alanine enrichment (MPE)	0.183 ± 0.025 ^##^	0.259 ± 0.024 *** ^##^	0.123 ± 0.011 ^##^	0.233 ± 0.016 *** ^##^
Posttraining				
Plasma (APE)	0.695 ± 0.039	0.663 ± 0.027	0.640 ± 0.016	0.638 ± 0.022
U leg [^2^H]-alanine enrichment (MPE)	0.469 ± 0.068	0.571 ± 0.067 ***	0.386 ± 0.024	0.474 ± 0.029 ***
T leg [^2^H]-alanine enrichment (MPE)	0.433 ± 0.050 ^#^	0.567 ± 0.059 ***	0.357 ± 0.026 ^#^	0.502 ± 0.036 ***

Values represent means ± SE. A two- and three-way mixed model ANOVA was used to investigate changes in plasma ^2^H and myofibrillar protein-bound [^2^H]-alanine enrichment, respectively. ***P < 0.001 significant increase from 0 h. ##*P* < 0.01, #*P* < 0.05 significant difference to U leg. APE, atom percent excess (%); MPE, mole percent excess (%); PLA, maltodextrin placebo group; PPB, postexercise and prebed protein-polyphenol group; T, trained leg; U, untrained leg.

Mean myofibrillar protein-bound [^2^H]-alanine enrichments are shown in Table 4. Over the pretraining acute measurement period, enrichments increased by 0.086 ± 0.009 and 0.095 ± 0.008 MPE in U and T legs (time effect *P* < 0.001). Enrichments were significantly greater in T than U legs (PLA: 22 ± 8%; PPB 14 ± 4%; leg effect *P* < 0.01), and was unaffected by PPB. Posttraining, mean myofibrillar protein-bound [^2^H]-alanine enrichments increased by 0.098 ± 0.010 and 0.131 ± 0.015 MPE (time × leg interaction *P* < 0.05), with enrichment at 0 h greater in U than T (post hoc *P* < 0.05). No additional effects of PPB were observed.

**Figure 4.**
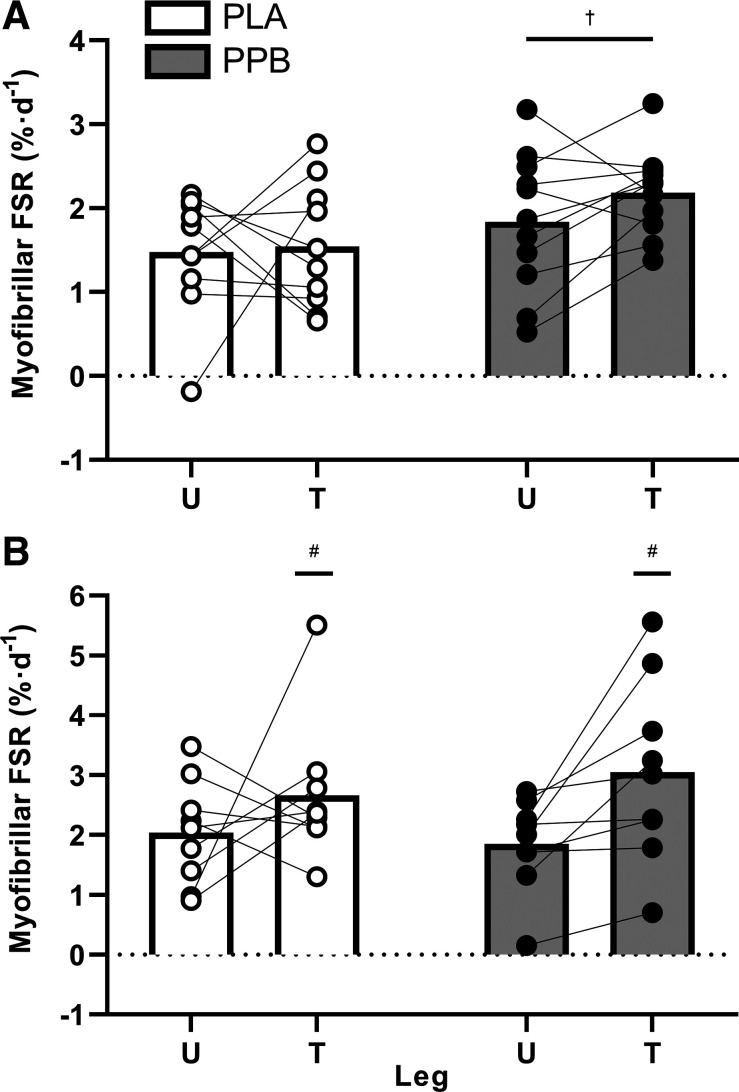
Free-living, cumulative myofibrillar protein fractional synthesis rate (FSR; expressed as %·day^−1^) over 48 h following the first (*A*) and final (*B*) session of unilateral resistance-type exercise in the trained (T) and untrained (U) legs. A postexercise and prebed protein-polyphenol (PPB; filled symbols; pretraining, *n* = 11; posttraining, *n* = 9) or maltodextrin placebo (PLA; open symbols; pretraining, *n* = 10; posttraining, *n* = 9) nutritional intervention was consumed daily. Fractional synthetic rates calculated from plasma deuterium enrichment as the precursor pool. Data are means ± SE. Statistical analysis performed with separate two-way ANOVAs. Significant main effect of group, denoted by †*P* < 0.05 significantly different to PLA. Significant main effect of leg, denoted by #*P* < 0.05 significantly different to U.

Plasma deuterium enrichment was used as the precursor pool to calculate daily myoFSR from the change in myofibrillar protein-bound [^2^H]-alanine enrichment during the acute measurement windows pre and posttraining. Pretraining (Fig. 4*A*), pooled myoFSR was 1.51 ± 0.16%·day^−1^ across legs with PLA. This was significantly greater with PPB, increasing to 2.01 ± 0.15%·day^−1^ (group effect *P* < 0.05). However, no differences were observed between U and T legs. Posttraining (Fig. 4*B*), PPB did not change myoFSR (*P* = 0.799). In contrast, the 31st training session increased myoFSR in T leg versus the rested U leg from 2.03 ± 0.29 to 2.66 ± 0.39 in PLA and from 1.85 ± 0.26 to 3.05 ± 0.51%·day^−1^ in PPB, respectively (leg effect *P* < 0.05).

### Whole Muscle Volume and Cross-Sectional Area

**Figure 5.**
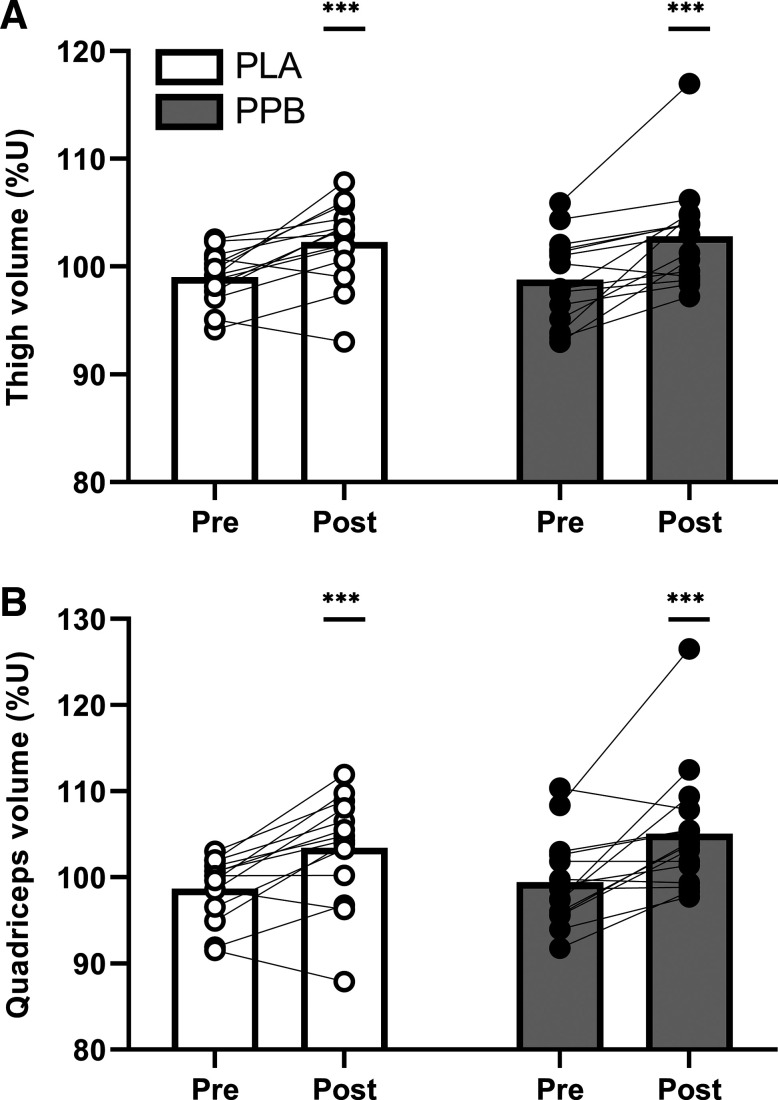
Whole thigh (*A*) and quadriceps (*B*) muscle volume measured using MRI pre and post 30 sessions of unilateral, maximal, isokinetic contractions, expressed relative to the untrained leg (%U). Postexercise and prebed protein-polyphenol (PPB; *n* = 15; filled symbols) or isocaloric maltodextrin placebo (PLA; *n* = 14; open symbols) nutritional interventions were ingested daily throughout the training period. Data are means ± SE. Statistical analysis performed with separate two-way ANOVAs. ***P < 0.001, significant main effect of training.

Thigh muscle volume increased from 99.0 ± 0.7 pretraining to 102.3 ± 1.0%U posttraining in PLA (time effect *P* < 0.001; Fig. 5*A*). There was no additional effect of PPB during training (98.8 ± 1.0 to –102.8 ± 1.2%U). Training also increased quadriceps muscle volume (*P* < 0.01; Fig. 5*B*) from 98.7 ± 1.0 to 103.4 ± 1.7%U in PLA and with no additional effect of PPB (99.4 ± 1.3 to –105.0 ± 1.9%U) pre to posttraining, respectively.

**Figure 6.**
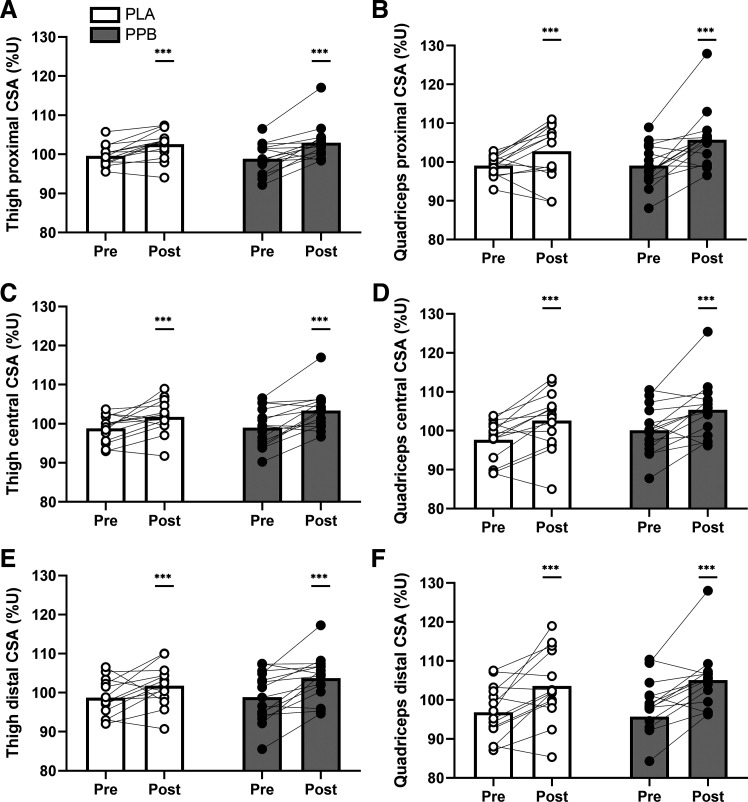
Whole thigh (*left*) and quadriceps (*right*) muscle CSA at proximal (*A* and *B*), central (*C* and *D*), and distal regions (*E* and *F*) measured using MRI pre and post 30 sessions of unilateral resistance-type exercise training, expressed relative to the untrained leg (%U). Postexercise and prebed protein-polyphenol (PPB; filled symbols; *n* = 15) or isocaloric maltodextrin placebo (PLA; open symbols; *n* = 14) nutritional interventions were ingested daily throughout the training period. Data are means ± SE. Statistical analysis performed with separate two-way ANOVAs. ***P < 0.001, significant main effect of training.

Training increased thigh muscle CSA at proximal (*P* < 0.001), central (*P* < 0.01), and distal regions (*P* < 0.01; Fig. 6). Quadriceps muscle CSA increased with training at proximal, central, and distal regions (all *P* < 0.01; Fig. 6). Intervention with PPB did not influence these changes.

### Muscle Fiber Characteristics

**Figure 7.**
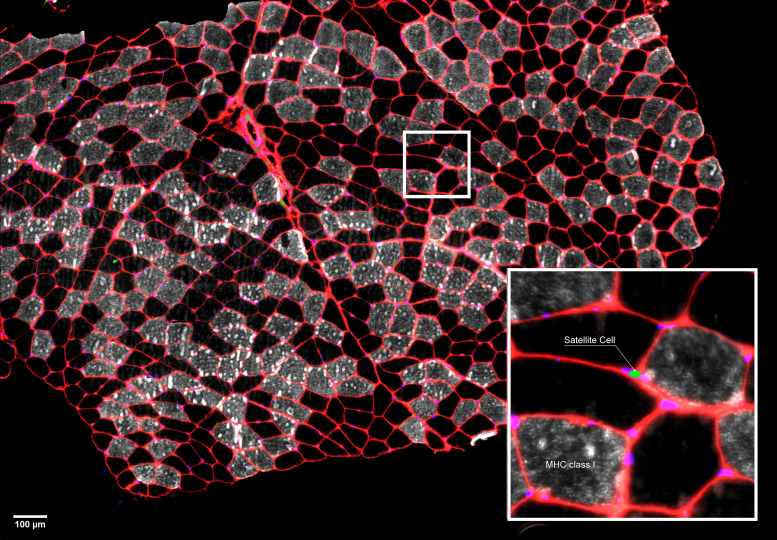
Representative composite image of a muscle cross-section stained for MHC-I (gray), laminin (red), and nuclei (DAPI; blue), with Pax7 (green) staining overlaid. The boxed area is magnified in the *bottom right* displaying one Pax7+ nuclei (SC). SC, satellite cell.

Due to limited remaining tissue, immunohistochemical analyses of muscle samples were determined in *n* = 9 for both groups. A representative image is shown in Fig. 7. Pretraining, the proportion of fibers that were type II was similar between U and T legs in PLA (61 ± 3% and 55 ± 3%, respectively) and PPB (64 ± 2% and 61 ± 5% in U and T, respectively). Posttraining, the proportion of type II fibers was unchanged (PLA: 58 ± 5% and 59 ± 5%; PPB: 57 ± 4% and 61 ± 3%; U and T, respectively; all comparisons *P* > 0.05).

**Figure 8.**
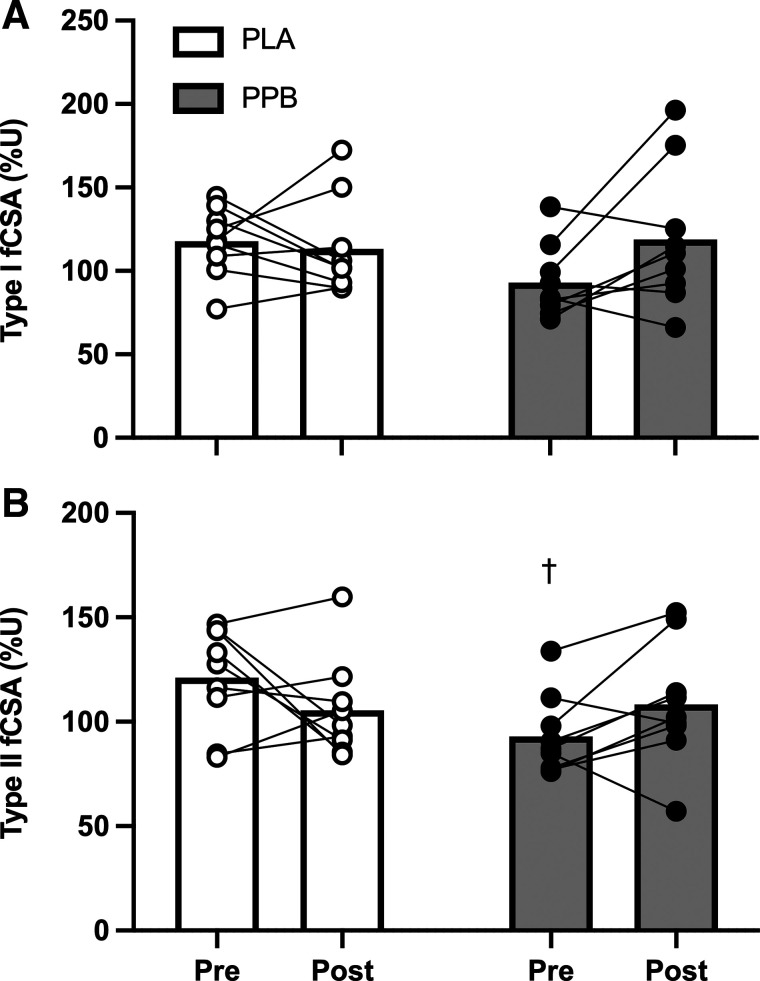
Muscle fiber cross-sectional area (fCSA) of type I (*A*) and type II (*B*) muscle fibers measured by immunohistochemistry pre and post 30 sessions of unilateral resistance-type exercise training, expressed relative to the untrained leg (%U). A postexercise and prebed protein-polyphenol (PPB; filled symbols; *n* = 9) or maltodextrin placebo (PLA; open symbols; *n* = 9) nutritional intervention was provided daily. Values are means ± SE. Statistical analysis performed with a two-way ANOVA. Significant time × group interaction effect (*P* < 0.05). Post hoc differences displayed as †*P* < 0.05 significantly different to PLA pretraining.

Mean fCSA was 120.5 ± 7.4 and 109.5 ± 8.6%U pre and posttraining in PLA. This change was influenced by PPB, such that fCSA was 91.5 ± 6.1 and 111.8 ± 10.7%U pre and posttraining (time × group interaction *P* < 0.05). Type I fiber size was unaffected by RET and by PPB intervention (Fig. 8*A*). In PLA, mean type II fiber size was 120.8 ± 8.2%U pretraining and 105.0 ± 7.9%U posttraining. However, PPB was 92.8 ± 6.2%U pretraining and 108.4 ± 9.7%U posttraining (Fig. 8*B*; time × group interaction *P* < 0.05).

The number of satellite cells per fiber pretraining was 0.020 ± 0.007 and 0.027 ± 0.008 SC·fiber^−1^ in U and T legs in PLA. This was unaffected by training at 0.034 ± 0.008 and 0.024 ± 0.008 SC·fiber^−1^ in U and T, respectively. This response was similar to PPB at 0.019 ± 0.006 and 0.014 ± 0.004 SC·fiber^−1^ in U and T legs pretraining, to 0.025 ± 0.006 and 0.028 ± 0.008 SC·fiber^−1^ in U and T legs posttraining (all comparisons *P* > 0.05).

### Muscle Protein and DNA Content

There were no differences in muscle protein and DNA content pretraining between groups or between legs (Table 5). RET did not influence muscle protein and DNA content, and no differences were observed posttraining. Similarly, there were no differences in protein:DNA ratio at any time point.

**Table 5.** Protein and DNA content of muscle samples collected pre and posttraining

	PLA		PPB	
	U	T	U	T
Pretraining				
Protein content, wet	591.3 ± 78.0	661.0 ± 60.2	777.1 ± 52.3	616.8 ± 73.7
DNA content, wet	1.5 ± 0.3	1.3 ± 0.2	1.3 ± 0.1	1.2 ± 0.2
Protein:DNA ratio	562.0 ± 77.1	530.7 ± 66.5	668.4 ± 60.1	474.8 ± 74.7
Posttraining				
Protein content, wet	687.8 ± 78.2	692.2 ± 70.2	789.4 ± 42.7	743.3 ± 52.5
DNA content, wet	1.5 ± 0.2	1.6 ± 0.3	1.3 ± 0.1	1.3 ± 0.1
Protein:DNA ratio	548.2 ± 83.2	585.2 ± 96.5	612.5 ± 51.9	602.8 ± 59.7

Values represent means ± SE. PLA, maltodextrin placebo group; PPB, postexercise and prebed protein-polyphenol group; T, trained leg; U, untrained leg; wet, µg/mg wet weight muscle tissue.

### Correlational Analyses

**Figure 9.**
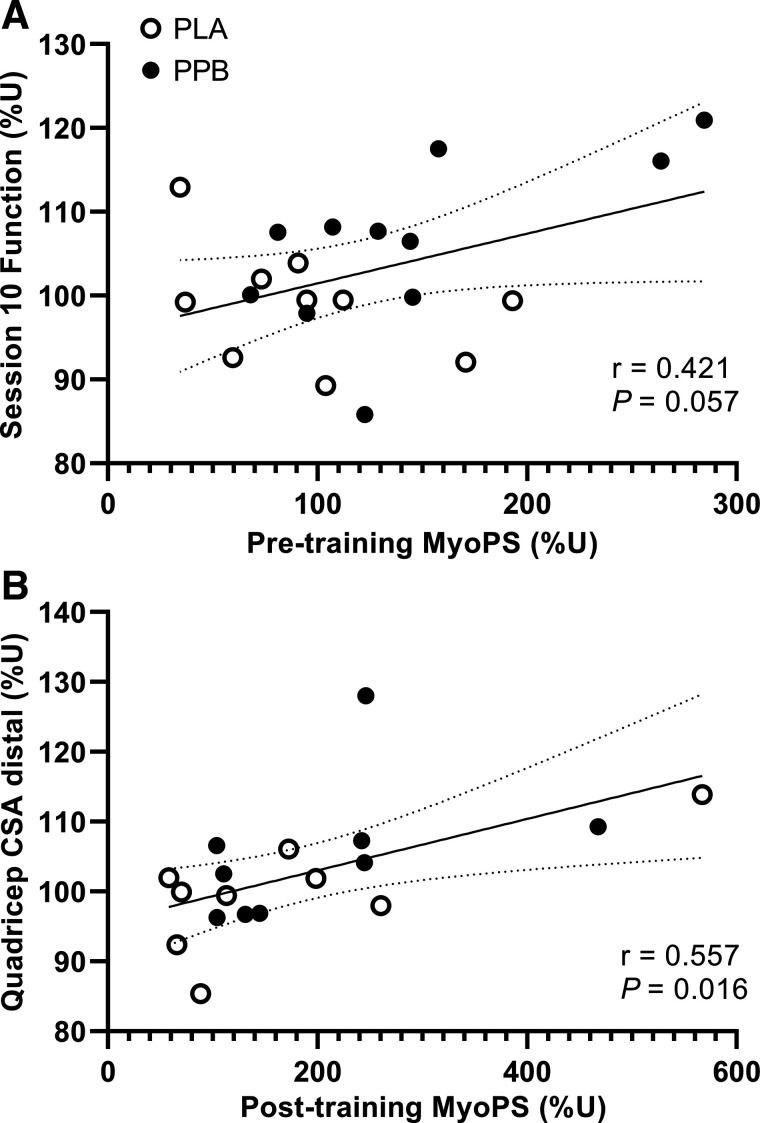
Correlations between 48-h myofibrillar protein synthesis rate expressed relative to untrained leg (%U) measured. *A*: pretraining, vs. knee extensor muscle function measured after 10 training sessions (*n* = 21). *B*: posttraining, vs. quadriceps cross-sectional area (CSA) at distal region (*n* = 18). Correlations performed with groups pooled, but open and filled circles represent PLA and PPB groups for illustrative purposes. Data analyzed by Pearson’s correlation analyses. Pearson’s *r* and *P* value displayed on each graph. PLA, maltodextrin placebo; PPB, postexercise and prebed protein-polyphenol.

There was a trend for myoFSR pretraining to be positively associated with muscle function at *session 10* (*r*^2^ = 0.18, *P* = 0.057; Fig. 9*A*). However, there were no relationships observed with any posttraining variable (Table 6). Conversely, myoFSR measured posttraining was positively associated with posttraining quadriceps CSA at the central (*r*^2^ = 0.23) and distal (r^2^ = 0.31; Fig. 9*B*) regions (both *P* < 0.05). Furthermore, there was a trend for both type I (*r*^2^ = 0.20) and type II (*r*^2^ = 0.20) fCSA to be positively associated with myoFSR posttraining (both *P* < 0.07).

**Table 6.** Correlations between dependent variables measured pre, during, and posttraining

	Pretraining MyoPS(%U)		Posttraining MyoPS (%U)	
	*r*	*P*	*r*	*P*
Function S10, %U	0.421	0.057	−0.140	0.579
Function posttraining, %U	−0.050	0.830	−0.313	0.206
Quadriceps volume, %U	0.068	0.771	0.399	0.101
Quadriceps CSA; proximal, %U	0.016	0.946	0.314	0.204
Quadriceps CSA; central, %U	0.181	0.258	0.478	0.045
Quadriceps CSA; distal, %U	0.249	0.276	0.557	0.016
TI fCSA, %U	0.317	0.182	0.443	0.066
TII fCSA, %U	0.299	0.229	0.439	0.069

CSA, cross-sectional area; MyoPS, myofibrillar protein synthesis rate; %U, expressed relative to time-matched untrained control leg; S10, *session 10*; TI fCSA, type I fiber cross-sectional area; TII fCSA, type II fiber cross-sectional area.

### Individual Response Analysis

Individual responses as standard deviation were calculated from the standard deviation of change scores (41) and displayed in Table 7. The individual response to PPB on quadriceps CSA at the distal region was very large (1.07 standardized units), with 90% confidence limits ranging from moderate (0.33 standardized units) to very large (1.47 standardized units).

**Table 7.** Change scores from pretraining with individual responses as standard deviation

	Change Scores (Means ± SD)		Individual Responses [SD (90% Confidence Limits)]
	PLA	PPB	
Function S10	−0.16 ± 1.27	0.63 ± 0.89	−0.91 [−1.41, 0.58]
Function posttraining	0.70 ± 1.57	1.17 ± 1.72	0.70 [−1.39, 1.71]
Quadriceps volume	1.05 ± 0.96	1.24 ± 1.32	0.90 [−0.65, 1.43]
Quadriceps CSA;proximal	0.85 ± 1.34	1.55 ± 1.50	0.67 [−1.17, 1.50]
Quadriceps CSA;central	0.82 ± 0.86	0.88 ± 0.97	0.45 [−0.74, 0.98]
Quadriceps CSA; distal	0.62 ± 0.66	0.86 ± 1.23	1.04 [0.32, 1.43]
TI fCSA	−0.12 ± 0.98	0.61 ± 1.20	0.70 [−0.77, 1.25]
TII fCSA	−0.38 ± 0.96	0.34 ± 0.71	−0.65 [−1.04, 0.50]

Values presented as standardized (Cohen) units with thresholds of 0.1, 0.3, 0.6, 1.0, and 2.0 representing small, moderate, large, very large, and extremely large effects (41). Values in parentheses represent 90% confidence limits. CSA, cross-sectional area; SD, standard deviation; S10, *session 10*; TI fCSA, type I fiber cross-sectional area; TII fCSA, type II fiber cross-sectional area.

## DISCUSSION

This study hypothesized that a daily postexercise and prebed PPB nutritional intervention known to accelerate recovery from muscle damage and increase MyoPS would accelerate improvements in muscle function over an early period of RET (∼3 wk; 10 sessions). Given the previously demonstrated tight coupling of MyoPS with hypertrophy at 10 wk of RET, we also hypothesized that this early improvement would be associated with greater posttraining MyoPS rates [i.e., after the attenuation of damage, as demonstrated previously (9)], and a greater increase in quadriceps muscle volume and fCSA following 30 sessions of RET in healthy males and females. Here, we demonstrate that, corrected to the untrained leg, PPB increased muscle function by ∼7% over the early phase of training, while there was no change in function in the placebo group. Moreover, daily rates of MyoPS were ∼33% greater over 48 h after the first training session with PPB versus placebo, whereby a trend (*P* = 0.057; Table 6; Fig. 9*A*) for a moderate correlation was observed between daily rates of MyoPS after the first training session and muscle function at the end of the early phase (i.e., after 10 sessions; ∼3 wk). These data are the first to show that a protein-polyphenol beverage known to suppress muscle damage can increase MyoPS and accelerate early improvements in muscle function at the onset of resistance training. However, whether this strategy permits greater muscle protein accrual during RET is unclear; PPB increased type II fCSA during training and the change scores of the ∼6% versus ∼10% increase in the quadriceps CSA at the distil region in PLA versus PPB were suggested to be small, while demonstrating a very large effect of individual variation. However, the ∼5% increase in quadriceps muscle volume pre to posttraining was similar between groups. Interestingly, rates of MyoPS measured posttraining correlated with central and distal quadriceps CSA (Table 6; Fig. 9*B*) but were not influenced by PPB. Similarly, PPB did not further increase the improvement in peak isometric torque and muscle function measured posttraining, suggesting that although this nutritional strategy promotes MyoPS and early functional adaptation, it may not influence longer term training adaptations.

Approximately 60% of the gains in total training volume or 1-repetition max (1-RM) typically occur within the first 3–4 wk of a 6-wk (8), 8-wk (25) or 12-wk (42) resistance training program. In this study, peak isometric torque increased over the early phase only (∼3 wk; Fig. 3*D*), and the rate of increase in total work performed during training was sevenfold greater over the early than late phase (Fig. 2*C*). Interestingly, eccentric work increased ∼26% during this early period only (Fig. 2*G*), possibly reflecting early remodeling (43, 44) or neurological adaptation that is reportedly more prominent in untrained than trained individuals (45). Given the magnitude of increase in work performed during training and our supposition that damage may otherwise impair early adaptation, the greatest potential to improve peak torque and function could occur over this time. By investigating these changes over time with high resolution, we demonstrate for the first time that a protein-polyphenol nutritional intervention increases muscle function by ∼7% over an early phase of RET. Interestingly, PPB did not further increase peak isometric or isokinetic torque, in agreement with our previous work demonstrating a favorable effect of PPB on recovery of muscle function but not isokinetic torque after muscle damaging exercise (17). Indeed, protein-based interventions appear not to augment increases in maximum strength measured by 1-RM over 3–4 wk of RET (2, 46, 47), but increase performance gains in aerobic and anaerobic power by ∼9–14% following 4 (48) or 6 wk (49) of sport-specific judo and canoe training, respectively. Reasons for these differences are unclear, but rapid neurological adaptation may be prominent in the first 2–3 wk of training (50, 51) masking any influence of nutritional intervention on strength gains (2, 46). Moreover, functional testing as employed in this study may capture an element of aerobic and/or anaerobic adaptation (52), which was augmented in the short term by provision of exogenous protein (53). Although a mechanism underpinning this adaptation is yet to be established, the apparent cyclical nature of this change in PPB but not PLA (Fig. 3, *A* and *B*) suggests a physiological mechanism, which warrants future investigation.

Although meta-analyses indicate an effect of protein supplementation on strength gains following RET protocols lasting >6 wk (15, 16), interestingly, such meta-analyses also show no effect of protein on strength when assessed by isometric or isokinetic peak torque (15), which has been attributed to lack of congruity between strength assessment (i.e., isokinetic or isometric) and training modality (i.e., isotonic) (54, 55). To account for this, and as aforementioned, our protocol measured peak isometric and isokinetic torque every three sessions (approximately weekly) throughout training, and each training session consisted of maximum isokinetic contractions, so participants were presumably not naïve to these measures. Moreover, correcting to the untrained leg reduced interindividual variability in isometric and isokinetic peak torque by 26% and 47%, respectively. Nonetheless, we unexpectedly observed that PPB did not influence either variable after ∼10 wk of training. For this reason, it may be that peak torque assessments, employed by us to assess muscle strength with greater resolution than typically afforded by 1-RM testing, may represent a unique contractile challenge to the muscle that captures an aspect of strength that is unaffected by protein ingestion. Therefore, the reasons why muscle function but not peak torque are influenced by nutritional intervention, either during RET as presently shown, or during recovery from eccentric exercise (17, 22), require further attention.

Our previous work showed that PPB intervention accelerated recovery of muscle function over 7 days following maximal eccentric exercise (17, 22). Interestingly, and in line with other studies (18, 19), we also demonstrated that MyoPS was not further stimulated by PPB in the days following damaging exercise (17, 22), presumably due to excessive muscle protein turnover during repair in the placebo group. Nevertheless, MyoPS was ∼35% greater with PPB compared with placebo beyond 72 h after eccentric exercise, once recovery of function had occurred. This study partially supports these findings, as although PPB accelerated the improvement of muscle function over ∼3 wk following the onset of unaccustomed RET (Fig. 3*A*), this was associated with ∼33% greater rates of MyoPS over 48 h than versus placebo. Although we have not investigated systemic measures of muscle damage, this stimulation of MyoPS together with there being no decline in function or peak isometric and isokinetic torque following the onset of RET (Fig. 3), is perhaps not consistent with the presence of severe muscle damage at this time. Indeed, the relatively small increase in muscle pain observed in this study (8/100) is far below that normally observed in classic models of muscle damage [i.e., 40/100; (56)]. This was unexpected given that a similar volume of isotonic resistance exercise (i.e., 54–72 repetitions each of concentric and eccentric contractions, at 80% concentric 1-RM; versus 90 concentric and 60 eccentric maximal contractions in this study) reportedly induces considerable (i.e., 60/100) muscle soreness (9), but may be caused by the extent of damage induced by different loading patterns throughout the range of motion between isotonic and isokinetic contractions (57). Regardless of the cause, the PPB-mediated improvement in function over 10 sessions was therefore unlikely underpinned by supporting recovery from damage, but instead may be related to greater MyoPS (Fig. 9*A*) that is observed thereafter (17).

The suggestion that MyoPS is directed toward repair of damage rather than hypertrophy at the onset of RET is based partly on the lack of correlation between eventual training adaptations and initial rates of MyoPS (9–11). However, despite our data being inconsistent with evidence of muscle damage, we add further support to the notion that these variables are unrelated by correcting to the control leg to account for interindividual variance. Yet remarkably, and in agreement with work published elsewhere (9, 58), MyoPS measured posttraining was significantly associated with quadriceps CSA at both central and distal regions and displayed a trend for association with both type I and type II fCSA. It should be noted that there was one negative FSR calculated for one individual in this study, and indeed it is not uncommon for negative rates of MyoPS to be presented in the wider literature [e.g., Fig. 4 by van Vliet et al. (59)]. This is likely due to FSR being an indirect calculation of a biological event that is the product of several complex analytical measurements with inherent variability (e.g., myofibrillar amino acid extraction). However, by correcting against the control leg, we reduce some of this variability and increase the reliability of any correlations against direct measurements such as quadriceps and fiber CSA. As such, not only do these data support a refinement of muscle protein turnover during training as hypothesized by others (60) such that MyoPS may be closer associated with net balance in the trained state, we suggest that factors other than damage repair per se are responsible for the dissociation between initial MyoPS and eventual hypertrophy. For example, this study is the first to strictly clamp protein intake (at 1.2 g·kg BM^−1^·day^−1^) during the measurements of daily MyoPS and we successfully manipulated these rates with additional exogenous protein at the onset of RET (Fig. 4*A*). Thus, daily rates of MyoPS may instead be sensitive to total daily amino acid availability, as per our previous observations (17, 22). Although it is intriguing that PPB did not increase MyoPS posttraining despite otherwise identical experimental conditions, intrinsically labeled protein feeding methodology demonstrates that exogenous amino acids comprise only ∼10% of all amino acids synthesized in muscle in the postprandial state (61), and recent evidence shows that 8 wk of RET reduces the proportion of exogenous amino acids incorporated into myofibrillar proteins by ∼33% (62). The high rates measured posttraining (i.e., 3.05 vs. 2.19%·day^−1^, or 0.127 vs. 0.091%·h^−1^, for T leg with PPB post vs. pretraining, respectively) are perhaps unexpected in the context of stable isotope tracer infusions [typically 0.07–0.08%·h^−1^ postexercise with protein (24, 63)]. However, deuterated water labels tracee irrespective of its source [i.e., both endogenous and exogenous amino acids; (64)]. Thus, the high measured rates are consistent with a considerable endogenous contribution to MyoPS in the resistance trained state, which would not be detectable with stable isotope infusion. Indeed, amino acid appearance from whole body proteolysis is reportedly greater in the trained state (58), and our MyoPS rates following exercise in the untrained state were in agreement with work published previously [i.e., 2.19%·day^−1^ vs. 1.98%·day^−1^ by Holwerda, et al. (34)]. Nevertheless, further direct experimental evidence is required to verify this suggestion and explore the relationship to training adaptations.

Whole muscle volume and CSA were measured before and after 30 sessions of RET to quantify skeletal muscle hypertrophy, as volume measured at the end of the early training period (i.e., 3 wk) would likely be confounded by edematous swelling (12). Corrected to the contralateral control leg, which reduced interindividual variation by 33%, whole thigh and quadriceps muscle volume increased ∼4% and ∼5%, respectively (Fig. 5), in agreement with ∼4%–8% greater quadriceps muscle volume previously reported following both isotonic (65, 66) and isokinetic training (3). Similarly, thigh and quadriceps CSA increased with training, with the increase in quadriceps CSA qualitatively greater at distal (∼8%) than central (∼5%) and proximal (∼5%) regions. Previous work indicates that muscle hypertrophy is potentiated by postexercise whey protein (67) and prebed casein protein (42) ingestion, and that increasing protein intake from 1.4 to 1.8 g·kg BM^−1^·day^−1^ during training increases (central) quadriceps CSA by ∼14% (15). However, although postexercise and prebed PPB increased self-reported protein intake to ∼1.8 versus ∼1.3 g·kg BM^−1^·day^−1^ in the placebo group (Tables 2 and 3), and that the protein (∼20 g) and leucine (∼2 g) content of each beverage likely provided a maximal anabolic stimulus (68), there was no clear effect of PPB on muscle volume (Fig. 5) or CSA (Fig. 6). Notwithstanding the potential inaccuracy of self-reported diet data, this finding is somewhat unexpected both in the context of the general body of literature (15, 16) and when considering our hypothesis that increasing MyoPS would be associated with greater gains in muscle size. Yet, evidence exists that protein- and/or amino acid-based supplements do not augment increases in whole muscle volume or CSA during RET using single-limb models (69) or isolated muscle groups (70). Although these previous reports may be attributable to low contraction volume [i.e., 18–30 repetitions/training session (69)] or a suboptimal quantity of additional protein [i.e., ∼10 g/day (70)], our similar findings arose despite our unilateral training model using 150 repetitions per session and our intervention providing 38 g of additional daily protein. Moreover, the absence of a difference in posttraining muscle volume and CSA remains consistent with our posttraining measures of function, peak torques, and MyoPS. As the increase in training load was notably smaller during the latter versus earlier phase of training and did not differ between groups (Fig. 2), the anabolic stimulus afforded by training was likely maximal across groups during this latter phase. Indeed, we observed greater standard deviation of change scores with PPB on quadriceps CSA at distal regions (Table 7), indicating a very large effect of individual responses to PPB. Although we acknowledge the possibility that a relative dose of protein may have provided a more uniform anabolic stimulus between individuals, the initial increase in MyoPS with PPB may have promoted early gains in muscle size, which were then masked as training load plateaued and MyoPS converged.

We observed that PPB did increase type II fCSA of the *m. vastus lateralis* over the training period, as has been observed elsewhere following RET (42, 71–74). Interestingly, type II fCSA gains of ∼18%–26% have been reported with milk, soy, whey, or mixed protein interventions during RET without concomitant gains in strength versus a control group (72–74), as demonstrated presently, suggesting a possible role of protein in mediating specific type II fCSA gains. The increase in satellite cell content per myofiber is reportedly related to the increase in fCSA (75, 76). However, there was no increase in satellite cell content per myofiber over time, or between trained and untrained legs at any time point. Furthermore, we observed no effect of PPB on any of these parameters, suggesting that this effect was independent of satellite cell numbers, and otherwise supporting the suggestion that expansion of the satellite cell pool is not a requirement of muscle fiber hypertrophy in humans (77, 78). Although there was a tendency for post training rates of MyoPS to correlate with type I and II fCSA measured post training, myofibrillar proteins comprise of ∼70% wet muscle weight (79) and so synthesis of myofibrillar proteins per se may not underpin this change. Rather, high volume resistance exercise may induce sarcoplasmic hypertrophy, possibly through greater synthesis of glycolytic proteins (80) and/or expansion of the intracellular fluid pool size (81), which may be potentiated by PPB. Indeed, this latter suggestion is consistent with the absence of change in protein:DNA ratio (Table 5), suggesting that fluid shifts may account for some of the change in fCSA (as well as whole muscle volume and CSA). Nonetheless, despite samples being normally distributed, taking a stereological approach with regard to fCSA, and correcting all data to the untrained leg, we observe high variability in both measures. Indeed, recent work reported that fCSA coefficient of variation was ∼12% in repeated biopsy samples obtained over a 24-h period with resistance exercise (82). Future work testing these possible associations is therefore required.

By using a unilateral training model allowing for intra-individual time-matched control to reduce heterogeneity in individual responses by ∼40%, these data show for the first time that a nutritional strategy known to suppress muscle damage increased rates of MyoPS by ∼33% and accelerated improvements in muscle function by ∼7% over an early period of RET (∼0–3 wk). However, whether this strategy permits greater muscle protein accrual during RET is unclear, as despite inducing type II fiber hypertrophy, there was only a small effect of the ∼6% versus ∼10% increase in quadriceps muscle CSA pre to posttraining for PPB versus PLA, with a large effect for individual responses in PPB. Nevertheless, the improvement in strength and function measured posttraining was unaffected by PPB, suggesting that although this strategy promotes MyoPS at the onset of training and early adaptation, it may not influence longer term training adaptations to a repeated unilateral stimulus.

## SUPPLEMENTAL DATA

10.6084/m9.figshare.17892506 Supplemental Tables S1–S3: https://doi.org/10.6084/m9.figshare.17892506.

## GRANTS

This work was part of a PhD studentship grant supported by the University of Exeter to F.B.S. A.J.M. and D.R.A. are supported in part by National Institute of Aging Grant P30-AG024832. J.F. was supported via an National Institute for Health Research (NIHR) grant to the University of Exeter (CRF/2016/10027).

## DISCLOSURES

This work was performed as part of a PhD studentship grant supported by the University of Exeter and Beachbody LLC. F.B.S. has received payments as a member of the Beachbody LLC scientific advisory board. K.M. and N.A. were employees of Beachbody LLC. The results of this study are presented clearly, honestly, without fabrication, falsification, or inappropriate data manipulation. None of the other authors has any conflicts of interest, financial or otherwise, to disclose.

## AUTHOR CONTRIBUTIONS

G.F.P., T.S.O.J., N.A., C.R.M., B.T.W., and F.B.S. conceived and designed research; G.F.P., T.S.O.J., J.R.B., J.F., D.R.A., and F.B.S. performed experiments; G.F.P., T.S.O.J., J.R.B., D.R.A., and A.J.M. analyzed data; G.F.P., T.S.O.J., A.J.M., B.T.W., and F.B.S. interpreted results of experiments; G.F.P. and J.R.B. prepared figures; G.F.P. and F.B.S. drafted manuscript; G.F.P., T.S.O.J., J.R.B., J.F., D.R.A., A.J.M., N.A., B.T.W., and F.B.S. edited and revised manuscript; G.F.P., T.S.O.J., J.R.B., J.F., D.R.A., A.J.M., N.A., B.T.W., and F.B.S. approved final version of manuscript.
